# The Perception of Labor Control and Employee Overtime Behavior in China: The Mediating Role of Job Autonomy and the Moderating Role of Occupational Value

**DOI:** 10.3390/bs15050691

**Published:** 2025-05-17

**Authors:** Wei Dong, Yijie Wang, Tingting Zhao

**Affiliations:** Department of Sociology, Hohai University, Nanjing 211100, China; wangyj_73@hhu.edu.cn (Y.W.); 18269894716@163.com (T.Z.)

**Keywords:** overtime behavior, labor control, de-skilling, job autonomy, professional value orientation

## Abstract

While the transformation of and improvements in productivity are taking place under the guidance of new technologies, overtime work—which is still prevalent in the workplace—is simultaneously introducing substantial physical and mental burdens to workers. Based on baseline data from the China Labor Dynamics Survey (CLDS), we analyze employees’ willingness to work overtime and their overtime cognition and intensity using labor process theory. It is observed that skill control directly increases the probability of employees’ objective overtime work, mandatory overtime work, and unconscious overtime work; furthermore, de-skilling prolongs working hours while hiding the prevalence and blurring the boundaries of overtime work. De-skilling also results in reduced employee job autonomy and further extends overtime hours, increasing the probability of mandatory overtime. Bureaucratic control reinforces the relationship between de-skilling and voluntary overtime, unconscious overtime, and overtime intensity by fostering employees’ career development orientation. It is necessary to accurately grasp the characteristics of new technologies in the changing labor environment of the new era, strive to create an equal and open labor market, and respect and protect the legitimate rights and interests of workers.

## 1. Introduction

With the rapid development of artificial intelligence technology, social productivity levels have greatly improved, and the traditional labor process is also being differentiated under new machinery and equipment iterations. However, productivity and income level developments have not reduced long work hours and high-intensity work as expected, and overtime work has become a prevalent work phenomenon in the current labor market. Some companies even go so far as to brand overtime work with euphemisms like “wolf culture”, emphasizing the need for professional competitiveness in such narratives. Moreover, these professions implement the “996” system of working from 9:00 a.m. to 9:00 p.m. 6 days a week, for a total of about 72 h a week. These working time regimes far exceed the stipulations on working hours under China’s Labor Law.

While public opinion has focused on attacking the “996” system in the workplace, a large number of workers are still silently working overtime. Labor-intensive industries increase production by extending working hours and increasing work intensity, and workers also realize increased income by working overtime under a piece-rate system. Floating prices and output render the piecework system more flexible, and while it is ostensibly a fair system rewarding increased work with increased pay, it is essentially the result of implicit forced labor under the hegemony of factories (Lv & Chen, 2014). In addition, unlike urban white-collar employees, factory workers have a substantially lower awareness of working hours and insufficient overtime cognition; even if the daily working hours exceed 8 h, as long as they work within the factory’s prescribed working hours, they are not considered to be working overtime; moreover, in some occasions, there is no so-called overtime compensation for work outside the factory’s prescribed hours. Some factories often blur legal boundaries using terms such as “peak production season” and “voluntary overtime”. For example, workers at Foxconn’s Hengyang factory work more than 100 h of overtime per month during the peak production season, far exceeding the 36 h limit stipulated in Chinese law (Global Times, 2018), and the factory requires workers to sign a “Voluntary Overtime Work Cutting Letter” at the beginning of the month (Z. Li et al., 2012). The 2024 Workplace Satisfaction Index Research Report revealed that 25.6% of employees work over 10 h daily, with blue-collar sectors such as services, manufacturing, and transportation facing more severe overtime challenges—over 30% of workers in the manufacturing industry work 10+ hours a day (Zhilian Recruitment, 2025).

It can be observed that overtime is not only a widespread phenomenon in China, but that the types of overtime work are also differentiated among different worker groups. In addition to the differences in industry characteristics, young migrant workers work an average of 225 h per month, which is substantially higher than that of highly educated employees in terms of both working hours and overtime work intensities. Young workers with no college education, foreign status, and agricultural household registration are more likely to be “overtime-dependent”, having to rely on overtime work to earn higher wages (Sun & Huang, 2021). Working overtime does not result in reasonable income compensation, and when flexible working is combined with performance-oriented work, the amount of unpaid overtime increases (Chung & van der Horst, 2020). This also indicates that salary and benefits are not the root cause of the current prevalence of overtime work; rather, it stems from more complex institutional and cultural factors. From the perspective of labor process theory, it is crucial to investigate the underlying causes of overtime practices.

Given the prevalence and complexity of overtime behavior in the current labor market and its rich research value, in addition to the blurring of the boundaries between work and life in the digital age (Geng et al., 2021), the study of overtime work and its effects has received substantial attention from the academic community. From the perspective of labor process theory, this study explores the effects of labor process control on specific dimensions of overtime behavior, such as the willingness to work overtime, overtime cognition, and overtime intensity.

## 2. Theory and Hypothesis

While existing research on overtime work has yielded substantial scholarly achievements, the new era necessitates an exploration of emerging characteristics in overtime practices and the formulation of corresponding research hypotheses for this study.

### 2.1. The Concept and Extension of Overtime Work

The concept of “overtime” essentially means “increased” work. Overtime refers to extended or increased working hours beyond the standard working hours stipulated by the state; this includes working hours on rest days and the time spent on commutes that take place earlier or later than the standard working hours (Shen, 2011). The current mainstream legal working hours are as follows: 75 countries, including China, adopt the standard 40 h workweek (i.e., 5 days working 8 h); 44 countries have legal working hours of 48 h or more. Overtime work is defined by legal working hours, and work is considered to be overtime work when an employed person is scheduled to continue to perform their job outside of legal working hours.

Globally, the attributions for overtime work can be categorized into two types. First, overtime occurs when workers cannot earn sufficient wages to make a living within statutory working hours. In classical economics, the subsistence wage consists of the amount of wages needed to maintain one’s existence and raise one’s offspring (Smith, 1983). As the quality of the workforce and the wage level of privately owned enterprises in China have long been generally low, and because China’s Labor Law stipulates that overtime work can be subsidized by more than double the amount of normal working hours, many workers voluntarily work overtime. Nominally, overtime work is “voluntary”, but, in reality, factories require workers to sign a Voluntary Overtime Declaration at the start of each month. Workers who refuse not only lose overtime opportunities but may also face various forms of harassment on the production line (Higher Education Foxconn Research Group, 2010; Z. Li et al., 2012). Second, self-employed and elite employees in scarce positions tend to engage in self-labor surplus extraction. As self-employed individuals own the means of production, in order to obtain higher income levels, they often correspondingly need to have higher labor intensities and extended labor working times (Zhu, 2018).

An extension of the above discussion is as follows: If traditional assembly line factory forms still define the labor time in terms of production time, then with the development of the platform economy and the emergence of new employment forms—such as flexible employment—the identification and specification of labor time will become increasingly difficult. In this context, the traditional concept and connotation of overtime work can no longer be fully applied, and overtime work should not only include the absolute number of hours worked outside the legal working hours but also take into account relative factors, such as the employee’s perception of the intensity of work or overtime work (Beckers et al., 2008).

Previous studies have usually only focused on overtime work, but they neglect the subjective worker’s perception of overtime work, which is manifested both in the voluntariness of overtime work and in their perception of overtime work. The so-called “voluntary overtime” is apparently a voluntary behavior but, in fact, it is coerced and inflicted via compulsion, which is shaped by a series of factors, such as labor processes, market pressure, enterprise systems, and the bargaining power of the workers (Jia & Zhong, 2018; Q. Li & Liu, 2020).

Additionally, few scholars have studied overtime cognition, which refers to a worker’s awareness of the fact that they work overtime. The concept of “afterhours work connectivity behavior” includes behaviors such as the use of smart mobile devices for work during non-working hours; in this scenario, the employee thus enters a state of “unconscious overtime”, during which the employee does not psychologically recognize that they are working overtime (Katherine & Cynthia, 2012). Unconscious overtime may occur either because employees confuse the on-duty and off-duty periods, or because they have an ambiguous perception of the number of hours they work. This study not only examines overtime work, but also the differences in employees’ perceptions of overtime work (i.e., what types of employees are more prone to unconscious overtime work).

### 2.2. Labor Control and Overtime Behavior

In *Das Kapital*, Marx centered his analysis on the labor process under capital control, where means of labor and objects of labor—key components of any productive activity—function inherently and continuously, regardless of specific conditions or temporal frameworks (Xie, 2007). In labor process theory, capital’s control over workers’ time is achieved through de-skilling. Mechanization results in machines substituting for workers’ physical labor, while automation leads to machines replacing workers’ skills—advances in production technology thus implicitly heighten the risk of unemployment for “versatile workers” in the labor market (Marx & Engels, 1972).

Braverman argues that the technological control of labor processes via management occurs not only at the level of physical technology, but also at the level of management technology. Under the advancement of standardized management and assembly line production, the most profound consequence has been the de-skilling of labor—a process where workers’ control over the labor process steadily erodes, leading to the disintegration of traditional production organizational forms. Braverman termed this the separation of “conception” and “execution” in labor processes, a dynamic that causes workers’ skills to deteriorate progressively and reduces them to fragmentary workers performing narrowly specialized tasks (Braverman, 1978). Through the separation of “execution” and “concept”, employers take away the autonomy of workers’ work (You, 2016).

While Marx and Braverman explained capital-enforced labor overtime well, Edwards’ “bureaucratic control” and Burawoy’s “hurry-up game” provided the theoretical basis for the study of voluntary overtime. Edwards proposed technical and bureaucratic control, where technical control directs the entire company’s workforce to a common work rhythm and work pattern determined via production technology, turning the employee group into homogeneous, unskilled, or semi-skilled operatives, while bureaucratic control controls the labor process with impersonal rules and procedures, setting up a system of rewards and penalties, corporate goals, and a culture within the firm (Edwards, 1979). Burawoy also argued that previous theories of labor control left no room for “consent” and observed that, over time, the capital’s control of the labor process shifted from one of coercion and fear to one of organization by consent, resulting in a situation in which “workers continue to work hard in the absence of coercion.” In the absence of coercion, “laborers continue to work hard to accomplish their tasks” (Burawoy, 2008), which comprises a landscape of self-exploitation.

Against the backdrop of great digital technology developments, labor differentiation has further intensified. On one hand, high-level mental labor and complex innovative labor are in short supply, while on the other hand, a large number of manual labor and transactional repetitive labor tasks have been marginalized (Q. Li, 2021). The skill level and competence of employees are key variables influencing their occupational status and wage levels. The distinction between high-skill and low-skill workers is primarily based on the varying skill requirements of different occupational types. Workers with higher competence not only command higher wages but also enjoy more comfortable working conditions and shorter working hours, while those with lower competence are more likely to be engaged in long-hour, low-return jobs (Guo, 2022). Workers with varying skill levels exhibit differences in both their overtime practices and perceptions of such work. Driven by survival logic, de-skilled workers are compelled to work longer hours to earn a higher income (Cai & Shi, 2016).

“De-skilling employees” refers to the work process in which the skills originally possessed by the employee are gradually weakened, simplified, or replaced, resulting in limitations imposed on their skill levels and career development. Knowledge and technology in production are transferred to engineers and managers who design the production process, and workers’ labor control and skill level are greatly weakened (Xu & Ye, 2020). The main definition of “de-skilled employees” in this study is as follows: in a capitalist-dominated labor process, technological control creates a division of the workforce, which causes de-skilled workers to become involved in the race for time on one hand and lose their autonomy in controlling their work on the other hand; this loss of autonomy exacerbates the capitalist control of the labor process, especially the control of labor time. This study will examine how “de-skilling”, “loss of worker autonomy”, and “bureaucratic control” in the labor process influence workers’ overtime behavior.

In labor control theory, scientific management breaks down complex labor into simple tasks, leading to de-skilling (Braverman, 1978). De-skilled positions, characterized by simplistic work content and high substitutability, are more likely to be categorized as “mandatory overtime” roles. Meanwhile, the dual labor market theory posits that de-skilled employees, confronting lower wages and benefits, often lack the capacity to reject mandatory overtime (Doeringer & Piore, 1971).

Human capital theory identifies skills and knowledge as core components of workers’ human capital, significantly influencing their market value and bargaining power (Becker, 1964). From the lens of resource conservation theory, individuals with scarce resources, like skills, are compelled to accept unfavorable conditions to avoid further losses—for example, enduring mandatory overtime to mitigate unemployment risks (Hobfoll, 1989). Social exchange theory argues that employees trade labor for compensation and security, with low-skill workers more prone to accepting adverse terms such as mandatory overtime (Blau, 1964).

De-skilling in the labor process reduces complex labor to simplistic tasks, making de-skilled workers highly replaceable, earning lower wages, and weakening their bargaining position against capital (Harilal, 1989; Robinson & Barron, 2007). When enterprises enforce overtime, de-skilled employees are highly likely to comply. Based on this theoretical synthesis, Hypothesis 1a is proposed:

**Hypothesis** **1a.** *De-skilled employees are more likely to be forced to work overtime.*

De-skilling weakens workers’ control over the labor process, potentially leading them to unconsciously extend working hours while being dominated. Blurred boundaries between work and life, as noted by Ashforth et al. (2000), cause employees to unknowingly prolong work time, creating “unconscious overtime”—a phenomenon exacerbated by technologies like computers and mobile phones that make work perpetually accessible, objectively extending working hours.

From the perspective of self-determination theory (Deci & Ryan, 1985), de-skilled work’s monotony may deprive employees of a “sense of presence”, prompting them to fill psychological voids through unconscious overtime. Occupational burnout theory (Maslach, 2003) further explains that the mechanical repetition in de-skilled roles increases emotional exhaustion and powerlessness, making employees more likely to accept invisible overtime numbly or even perceive it as inherent to their duties.

This process involves a chain of reactions: skill devaluation from de-skilling reduces bargaining power, technology erodes work–life boundaries, and burnout diminishes resistance. Under de-skilling, institutional norms, algorithmic management, and psychological inertia collectively drive unconscious overtime. De-skilled workers, lacking marketable skills and clear awareness of employers’ scheduling strategies, face a heightened likelihood of such unrecognized overtime. Based on these cumulative factors, Hypothesis 1b is proposed:

**Hypothesis** **1b.** *De-skilled employees are more likely to work overtime without realizing it.*

In surplus value theory, due to de-skilled employees lacking specialized skills, capitalists tend to increase profits by extending working hours rather than investing in technology. De-skilled workers, deprived of control over production pace, are compelled to accept high-intensity overtime to meet quotas. Technological applications like automated equipment serve as tools for management to enhance control—de-skilled employees, reliant on standardized processes, have their work rhythms and intensities more easily manipulated by technical systems (Edwards, 1979). The combination of high work demands and low decision-making autonomy typically leads to employees enduring greater work intensity (Karasek, 1979).

In the secondary labor market, de-skilled workers—characterized by low wages, high turnover, and limited career prospects—are at a disadvantage in labor-capital negotiations. Enterprises use an “overtime-dependent system” to tie working hours to compensation, forcing workers to accept strenuous overtime. Technical control reinforces managerial dominance: standardized processes and algorithmic management facilitate working hour extensions. Research has shown that overtime duration in de-skilled positions is significantly longer than in high-skill roles (Autor et al., 2003), and longer overtime hours generally correlate with greater intensity. Based on this, Hypothesis 1c is proposed:

**Hypothesis** **1c.** *Overtime may be more intense for de-skilled employees.*

### 2.3. The Mediating Effect of Job Autonomy

The mediating variable in this study is job autonomy, a core condition for satisfying individuals’ intrinsic motivation and driving positive behaviors. Within the job demands-resources model (Demerouti et al., 2001), job autonomy functions as a critical resource that mitigates the negative impacts of work demands on employees while enhancing performance through increased work engagement. Employees with high levels of autonomy are more likely to proactively regulate their work pace and improve efficiency.

The classical mediating effect framework (Baron & Kenny, 1986) provides a methodological foundation for identifying how job autonomy acts as a mediating mechanism. Substantial research has established the mediating role of job autonomy across multiple theoretical perspectives:

Self-determination theory posits that autonomy is a core driver of intrinsic motivation, a fundamental psychological need. External factors such as leadership style and organizational support shape employee attitudes and behaviors by fulfilling autonomy needs (Ryan & Deci, 2000).

The job characteristics model (Oldham & Hackman, 1980) highlights that core job attributes—including skill variety, task significance, and autonomy—influence employee motivation and behavior through “psychological states” like perceived work meaning and responsibility. Among these, job autonomy directly impacts employees’ intrinsic motivation and sense of control over their work processes.

Empowerment theory identifies job autonomy as a central dimension of psychological empowerment (Conger & Kanungo, 1988; Spreitzer, 1995), mediating the relationship between empowerment practices (e.g., delegated decision-making) and positive employee outcomes such as enhanced engagement and performance.

This study, however, focuses on the mediating role of job autonomy in the relationship between de-skilling and overtime behavior. The proposition that “de-skilling influences overtime behavior by reducing job autonomy” is anchored in labor process theory, which posits that capital, through de-skilling, leads to passive compliance with enforced extended work hours. Braverman (1978) highlighted that the “separation of conception and execution” erodes workers’ control over labor processes, while Edwards (1979) argued that de-skilled employees under technical control—reliant on standardized procedures and lacking job autonomy—have their work rhythms and intensities directly manipulated.

From the perspective of social control theory, management employs de-skilling to diminish employee autonomy and enforce mandatory overtime. The job characteristics model (Hackman & Oldham, 1976) further underscores that low autonomy compels employees to passively accept work arrangements: de-skilled roles, characterized by task standardization and limited decision-making authority, leave workers unable to autonomously regulate their work hours. De-skilling in the labor process dismantles skilled workers’ sovereignty over their work—not only through physical technologies but also via managerial techniques of control. When employees lose autonomy in managing their work, the likelihood of mandatory overtime increases significantly. Based on this theoretical reasoning, Hypothesis 2a is proposed:

**Hypothesis** **2a.** *De-skilling forces employees to work overtime through de-autonomy.*

De-skilling leads to the mechanization of work content, stripping employees of autonomy over work pace and methods, thus making it difficult to control their own working hours. From the perspective of boundary theory, the penetration of information technology blurs the divide between work and life, preventing employees from autonomously switching contexts and leading to unconsciously extended work time (Ashforth et al., 2000). Self-determination theory (Deci & Ryan, 1985) emphasizes that in de-skilled roles lacking autonomy, employees may compensate for psychological deficits through unconscious overtime—a form of filling existential voids created by monotonous tasks. Resource conservation theory (Hobfoll, 1989) further posits that when resources like autonomy are scarce, individuals tend to over-invest in work to mitigate perceived losses, often resulting in unrecognized overtime.

Occupational burnout theory (Maslach & Leiter, 2022) highlights that de-skilled jobs, characterized by high repetition and low autonomy, induce emotional exhaustion and powerlessness. Employees in such roles may unconsciously work overtime to compensate for inefficiencies stemming from stripped decision-making authority. As automation robs de-skilled workers of autonomous decision-making and enterprises use technology to blur work–life boundaries, unconscious overtime becomes normalized. Against this theoretical backdrop, Hypothesis 2b is proposed:

**Hypothesis** **2b.** *De-skilling enables employees to work unconscious overtime through de-autonomy.*

De-skilled workers, lacking control over production pace, are compelled to accept high-intensity overtime to meet output quotas. Management systematically reduces employee autonomy through de-skilling, stripping workers of the ability to dictate their work rhythms and methods. Low-skill employees, facing limited career development and skill-upgrading opportunities, often fall into a vicious cycle of “low autonomy-burnout-passive overtime”—forced to endure strenuous overtime to secure their jobs.

Technical control further exacerbates overtime intensity (Frenkel et al., 1995), alienating workers into mere tools in efficiency chains who sacrifice free time for maximized productivity (D. Wang, 2007). Deprived of job autonomy, de-skilled employees become passive implementers of corporate efficiency goals. Against this backdrop, Hypothesis 2c is proposed:

**Hypothesis** **2c.** *De-skilling extends employee overtime through de-autonomy.*

### 2.4. The Moderating Effect of Career Value Orientation

The moderating variable in this study is career value orientation. Career development theory (Super, 1957) categorizes career value orientation into multiple dimensions: existence orientation (salary, job stability), development orientation (skill enhancement, career growth), intrinsic value (work meaning, autonomy), and extrinsic value (status, recognition). An individual’s career value orientation shapes their perception of the work environment, thereby moderating the relationship between external factors and employee behavior.

The moderating variable framework (Baron & Kenny, 1986) defines moderating variables as factors that affect the direction or strength of the relationship between independent and dependent variables. Numerous studies on the moderating effect of career value orientation have shown that employees with a strong existence orientation—prioritizing income and job stability—exhibit higher tolerance for overtime in repetitive roles, while those with a strong development orientation—valuing skill enhancement and career growth—demonstrate significantly lower willingness to work overtime due to unmet developmental needs (Chen et al., 2018).

Employees driven by existence orientation focus on immediate income stability, potentially using overtime to compensate for reduced career security caused by skill devaluation. In contrast, development-oriented employees may weaken the positive association between de-skilling and overtime by reducing overtime participation and seeking training opportunities (Kalleberg, 2018).

When acting as a moderating variable, different dimensions of career value orientation (existence vs. development) alter the mechanism through which the independent variable (de-skilling) affects the dependent variable (overtime behavior):

For existence orientation, in contexts of resource scarcity or high career risk, this orientation strengthens individuals’ reliance on economic security, leading them to accept high-intensity overtime or low-skill jobs as a survival strategy.

For development orientation, when individuals prioritize career growth or social value, they proactively offset the negative impacts of de-skilling through skill enhancement or career (career reinvention), weakening the causal link between de-skilling and overtime behavior.

Career values shape individual occupational behavior (Super, 1957). Employees with strong development orientation values prioritize long-term career growth, perceiving de-skilling as a short-term challenge and leveraging learning opportunities during overtime to overcome professional plateaus. This orientation enhances their sensitivity to resource loss, prompting them to prioritize protecting career development resources in de-skilled contexts—thereby reducing inefficient overtime.

Boundary theory (Ashforth et al., 2000) posits that blurred work–life boundaries lead to unconscious overtime, yet employees with high development orientation—who value work meaningfulness—actively set work boundaries to avoid excessive hours, moderating the relationship between de-skilling and overtime. By strengthening the pursuit of work meaning, development orientation values encourage employees to maintain work–life balance even under de-skilling pressures.

Human capital theory (Becker, 1964) identifies skills as core labor competitiveness. Driven by career development values, individuals invest in skills to enhance market bargaining power: those with strong development orientation may view overtime as a means of human capital accumulation rather than mere time expenditure, integrating it into strategic career planning.

Employees with strong development orientation values transform de-skilling into resource accumulation opportunities through skill learning during overtime. Driven by career development needs, they actively reshape job content, converting passive overtime into proactive skill enhancement. Such values also guide them to choose roles aligned with long-term growth, avoiding inefficient overtime in de-skilled positions.

Technical control pushes employees into passive overtime, while bureaucratic control—by optimizing management practices and fostering organizational culture—shapes their career values to moderate overtime behavior. High-skill employees, possessing core knowledge, higher hierarchical status, and greater work autonomy, often identify with organizational goals, viewing overtime as a means to pursue promotion and self-actualization (Yan, 2020; Liang, 2019; Hou & He, 2020; Zheng et al., 2021). Based on this, Hypothesis 3a is proposed:

**Hypothesis** **3a.** *Development-oriented value plays a moderating role in de-skilling and overtime behavior.*

Resource conservation theory (Hobfoll, 1989) posits that employees with strong existence orientation values treat economic security and job stability as core resources. When de-skilling threatens these resources, they may proactively extend working hours to compensate for skill deficiencies and safeguard job stability. By enhancing sensitivity to resource loss, existence orientation values drive individuals in de-skilled contexts to prioritize overtime as a strategy for preserving survival resources.

Within the job demands-resources model (Bakker & Demerouti, 2007), employees with high existence orientation place greater emphasis on survival resources, using overtime to mitigate resource shortages. This orientation increases their reliance on economic rewards, making them more inclined to trade overtime for financial compensation rather than pursue autonomy or career development in de-skilled scenarios. Low-skill workers, in particular, may accept “unconscious overtime” out of unemployment anxiety, viewing extended hours as a necessary trade-off for maintaining job security.

Employees with strong existence orientation values, when faced with de-skilling, tend to sacrifice long-term development for short-term stability, accepting repetitive tasks and extended working hours. De-skilling diminishes employees’ human capital value, while existence orientation amplifies their economic reliance on overtime—a trade-off logic where “overtime secures survival” emerges from the combination of low bargaining power in de-skilled roles and basic subsistence needs. This value orientation may suppress the pursuit of skill enhancement, driving individuals to prioritize overtime as an economic safeguard instead.

The interaction between de-skilling and existence orientation creates a low-skill-high-overtime vicious cycle: employees with high existence orientation treat economic security as a core resource, prioritizing overtime to maintain income when de-skilled. Employees with high existence orientation may tacitly accept overtime due to financial pressures, normalizing it as a “routine” work pattern. Based on this, Hypothesis 3b is proposed:

**Hypothesis** **3b.** *Survival orientation values play a moderating role in de-skilling and overtime behavior.*

**Figure 1:**
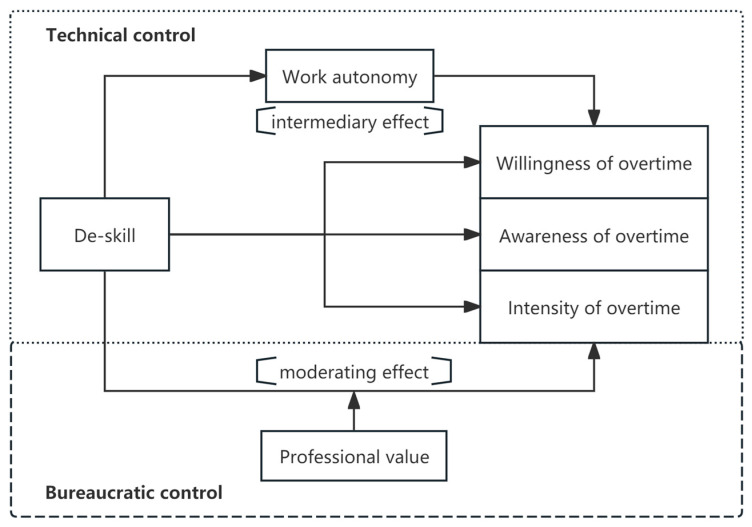
Conceptual model.

Figure 1 illustrates the main concepts and ideas of this study. Independent variable de-skilling refers to the weakening of employees’ skills in the work process. The overtime behavior as a dependent variable is subdivided into three dimensions: willingness to work overtime, overtime cognition, and overtime intensity. The willingness to work overtime is the psychological tendency of employees to work overtime, overtime cognition is the perception of employees in working overtime, and overtime intensity indicates the degree of overtime work relative to the length of overtime work. Work autonomy as a mediating variable refers to the extent to which employees can make decisions and organize their work methods and schedules. The occupational value as a moderating variable refers to the employee’s comprehensive evaluation of the fulfillment, social status, and financial rewards of their occupation. The technological control context emphasizes the control of the labor process through de-skilling, while the bureaucratic control context focuses on managerial control through institutions and hierarchical structures.

## 3. Data and Methods

Drawing on data from the China Labor-force Dynamic Survey (CLDS), this study operationalizes variables to analyze overtime behavior under labor control, employing multiple analyses of mediating and moderating effects to achieve a more scientifically rigorous research design.

### 3.1. Data Use and Variable Setting

This study uses survey data at the individual labor force level from the 2016 China Labor Dynamics Survey (CLDS2016), a large-scale interdisciplinary tracking survey that focuses on the current status and changes in China’s labor force. It covers a wide range of research topics, including education, work, migration, health, social participation, economic activity, and grassroots organizations. The survey’s sample covers 29 provinces and cities in China, ensuring a representative sample that can be better applied to analyze overtime behavior. Some scholars have pointed out that the CLDS reveals the transformational characteristics of China’s dual–dual labor market through a comparison of labor rights and interests inside and outside the compilation, and the tracking data also provide timely support for policy evaluation.

The CLDS2016 data contain rich labor market information, which is still valuable for the current study. As a tracking survey, the CLDS makes it possible to establish a time series analysis to observe long-term trends in labor market changes. Moreover, the theme of this study, “overtime work”, is still relatively common in the current social development context. Thus, it is necessary to review and analyze the data accumulation and social situation in the previous period in order to provide references for the current “overtime work” phenomenon. This study uses CLDS2016 data after comprehensive considerations.

As the topic of discussion is overtime work behavior, according to China’s Provisions on the Special Protection of Underage Workers, employers are required to have special protection measures in place for workers aged 16–18 (underage workers). The sample of the study is limited to non-agricultural employees aged between 18 and 64 years old, and after eliminating the missing values of various relevant variables, a total of 5263 samples were included in the analysis.

#### 3.1.1. Dependent Variables

Overtime behavior, as the dependent variable, was refined in this study into three dimensions: willingness to work overtime (attitude), perception of overtime (perception), and intensity of overtime (time).

In addition to asking respondents about actual overtime work, CLDS2016 also asked the following in the “Employee” section: “Do you usually work overtime voluntarily at your current workplace?” If the respondent answered “yes”, the variable was coded 1 for “voluntary overtime” and 0 for “mandatory overtime”, generating a variable for willingness to work overtime.

CLDS2016 also asked respondents in the “Employee” section, “Do you normally work overtime”? If the respondent answered “yes”, it was determined that there was “subjective overtime” behavior, which was coded as 1. When given the vice versa answer, the answer was coded as 0, and a subjective overtime variable was generated. Depending on the respondent’s “objective overtime” and “subjective overtime” status, if the respondent answered “yes” to the former and “no” to the latter, it was determined that the respondent exhibited “objective overtime” and “subjective overtime” behavior. If the respondents answered “yes” to “objective overtime” and “subjective overtime” and ”no” to “objective overtime” and “subjective overtime”, the respondents were recognized as participating in “unconscious overtime” and were “cognizant of working overtime”, where “unconscious working overtime” was coded as 1 and “cognizant of working overtime” was coded as 0 to generate the overtime cognitive variable.

Overtime refers to the extension of workers’ working hours beyond legal working hours. CLDS2016 asked the respondents, “how many hours per week do you usually work at your current or most recent job” under the “Worked in the past year” column, and if the number of the respondent’s answer exceeded 44, it was coded as 1; otherwise, it was coded as 0. If the respondent’s answer numbered more than 44, “objective overtime behavior” was considered to be present and coded as 1; otherwise, it was coded as 0, which generates the objective overtime variable. Based on the objective overtime code of “1”, the respondent’s answer was subtracted from 44 to obtain the overtime length (hours), which generates the overtime work intensity variable.

#### 3.1.2. Independent Variables

In exploring the causes of overtime behavior, labor de-skilling is the independent variable. In this study, we operationalized “technical control” as “labor de-skilling” by asking the following: “In your opinion, do you need to receive special training or training to do this job well?” The questionnaire also included the following: “In your opinion, does this job require any special training or training to do it well?” If the respondent answered “yes”, then there was no de-skilling in the job, which was coded as 0. If the respondent answered “no”, then there was de-skilling, which was coded 1. Moreover, the de-skilling variable was generated.

#### 3.1.3. Mediating and Moderating Variables

The mediating variable in this study is “work autonomy”, and the CLDS questionnaire sets the following questions: “To what extent do you decide the following things in your work” under the column of “Having worked in the past year?” The dimensions examined include “content of work tasks”, “organization of work schedule”, and “workload and intensity of work”. When the respondent answered “completely decided by myself”, it was coded as 3; “partially decided by myself” was coded as 2, and “completely decided by others” was coded as 1. The scores of the three dimensions were summed up to obtain the continuous variable for work. The three dimensions were summed to obtain a continuous job autonomy variable, with scores ranging from [3, 9]; the higher the score, the greater the job autonomy.

The moderator variable in this study was “occupational value orientation”. Under the “Worker Status” section, the CLDS questionnaire was designed to ask respondents about their value perceptions of their current job: “What is the meaning or value of your current job to you?” (If there is no current job, the question is about the most recent job.) The dimensions examined are as follows, in descending order: “earning a living”, “giving myself peace of mind”, “meeting more people”, “getting to know people”, “gaining respect”, “interests”, and “utilizing one’s abilities to the fullest”. The questionnaire provided five options for the above question: “very much in line”, “quite in line”, “indifferent”, “quite out of line”, and “very much in line”; these were coded on a scale of 1–5, respectively. Exploratory factor analysis yielded a “KMO value of 0.816”, indicating strong commonality among variables and suitability for principal component extraction (Zhuang, 2018). Finally, two factors were extracted, which were named “survival orientation factor” and “development orientation factor”. After polar variance analysis, “survival orientation factor” and “development orientation factor” were examined. After standardization, two standardized variables with values from 0 to 100 were generated, representing the two occupational value orientations of workers: “Survival Orientation” and “Development Orientation”. The higher the scores, the higher the degree of the orientation. The higher the score, the higher the worker’s orientation value.

In addition, other control variables are included in the model. The measurement and assignment of each variable are shown in Table 1.

### 3.2. Statistical Models

The dependent variable, overtime hours, is a continuous variable, and the linear regression model is specified as follows:

(1) $$y_{i}=\alpha_{0}+\alpha_{1}type+\varepsilon_{i}$$

Here, $y_{i}$ represents the overtime hours of employees, $\alpha_{0}$ is a constant term, $\alpha_{1}$ is the regression coefficient of the independent variable, type represents the de-skilling of the labor independent variable, and $\varepsilon_{i}$ is the random disturbance term.

The dependent variables, voluntary overtime and overtime cognition, are binary variables, and the binary logistic model is specified as follows:

(2) $$\ln\left(\frac{p}{1-p}\right)=\beta_{0}+\beta_{1}type+\varepsilon_{i}$$

(3) $$\ln\left(\frac{p}{1-p}\right)=\beta_{0}+\beta_{1}C_{S}+\beta_{2}C_{M}+\beta_{3}C_{F}+\beta_{4}C_{H}+\varepsilon_{i}$$

Here, p is the probability of voluntary overtime/unconscious overtime; 1−p is the probability of compulsory overtime/perceived overtime; type denotes de-skilled labor; $\beta_{1}$ represents the regression coefficient of the independent variable, indicating the degree of influence of the independent variable on voluntary overtime/unconscious overtime (if the value is positive, this indicates that the variable is a positive factor, and the greater the coefficient, the greater the degree of influence); $\beta_{0}$ denotes the constant term; and $\varepsilon_{i}$ denotes the random disturbance term.

**Figure 2:**
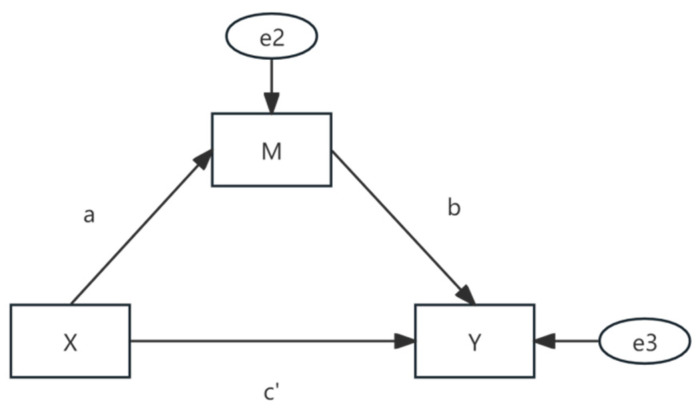
Principle of mediation effects.

In order to estimate whether the job autonomy degree plays a mediating role in the relationship between “labor de-skilling” and overtime work, as well as the size of the mediating effect, this study adopted the bootstrap method in the analysis to test the intermediary effect. As shown in Figure 2, the idea of the intermediary effect originates from the indirect effect. c is the total effect of X on Y; ab is the indirect effect through intermediary variable M; $c^{\prime }$ is the direct effect. Moreover, the regression coefficient meets $c=c^{\prime }+ab$. In this study, X indicates whether there is “de-skilling” in the labor process, M indicates the work autonomy of the employee, and Y indicates the overtime working time. The intermediary effect is used to test whether the ab effect exists and its proportion: that is, whether the “de-skilled” degree of employees in the labor process extends the overtime period of employees by reducing or improving their work autonomy.

In terms of testing the adjustment effect, this study adopts the most basic model, which is as follows:

(4) Y=aX+bM+cXM+e

Here, the coefficient c denotes the size of the adjustment effect coefficient, M denotes the adjustment variable, and XM denotes the interaction term between the independent and adjustment variables. As some of the adjustment variables in this study are continuous variables, decentralized processing is carried out on them. The specific formula is as follows:

(5) $$Y=aX+bM+c\left(X-\bar{X}\right)\left(M-\bar{M}\right)+e$$

## 4. Overtime Work Under Labor Control

In this study, overtime behavior under labor control is broken down into three dimensions for analysis: overtime willingness, overtime cognition, and overtime intensity. The effects of de-skilling—a specific form of labor control—on employees’ overtime behaviors are further observed through the mediating role of job autonomy and the moderating role of career value orientation.

### 4.1. Descriptive Statistics of Overtime Work Behavior

Table 2 shows the statistical results of the different orientations of overtime work. From an objective point of view, overtime work has become a common phenomenon among workers, with 52.24% of employees working more than 44 h per week. It can be observed that only 32.64% of employees believe that overtime work exists in their work, while most do not believe that there is overtime work. Among all the workers who worked overtime, more than 60% of them worked overtime unconsciously. This indicates that overtime work has not only become a common phenomenon in the current labor market but also that the employees’ awareness of overtime work is also generally insufficient; that is, the perception of overtime work can, in fact, reflect the employees’ perception of labor control, and the insufficient awareness of overtime work reflects the employees’ lack of awareness toward ambiguous labor control. The lack of overtime perception reflects the employees’ lack of awareness with respect to ambiguous labor control.

### 4.2. Technical Control and Employee Overtime

The “de-skilling” of the labor process results in “labor degradation”, and technological control is also an important means of time control by capital. Therefore, this analysis focuses on the impact of de-skilling on employees’ voluntary overtime work (willingness), unconscious overtime work (perception), and overtime hours (intensity).

#### 4.2.1. The Impact of Labor “De-Skilling” on Employees’ Willingness to Work Overtime

Table 3 presents the regression results of labor de-skilling on voluntary overtime. The baseline model in “Column 1” excludes the core independent variable “labor de-skilling”, including only employee demographic characteristics and firm-level attributes. The labor control model in “Column 2” adds the labor de-skilling variable to the baseline model, incorporating the theoretical construct of interest into the analytical framework.

From the regression results of the benchmark model, it can be observed that among the personal characteristics variables, only household registration and income play a significant role, while the rest of the variables do not have a significant effect on employees’ voluntary overtime. The probability of voluntary overtime work is lower for urban employees, with a 27.5% lower probability of overtime work for urban employees relative to rural employees (1 − e^−0.275^, *p* < 0.001). Those with a higher income are more likely to work overtime voluntarily. Among firm characteristic variables, the unionization and provision of room and board significantly affect the voluntary overtime behavior of employees. The existence of trade union organizations increases the compulsory overtime work probability of employees, and having a trade union in the enterprise decreases the probability of voluntary overtime work by 24.5% (1 − e^−0.245^, *p* < 0.05). The provision of room and board by the enterprise significantly increases the likelihood of employees working overtime voluntarily, with the probability of employees working overtime voluntarily increasing by 52% (e^0.419^ − 1, *p* < 0.01). On the surface, the “all-you-can-eat, all-you-can-live” dormitory labor system provides convenience for employees and allows them to rest easily but, in reality, the employer extends the working hours of the workers by controlling their living space and makes them work overtime in an implicit manner.

The labor control model is based on the baseline model with the addition of the labor de-skilling variable, and, as observed in the regression results, the significance of and coefficient changes in the control variables are low compared to the baseline model. Moreover, the labor de-skilling independent variable plays a significant role in the voluntary overtime of employees. Compared to employees who are not de-skilled at work, the probability of carrying out voluntary overtime work for employees who are de-skilled at work is significantly lower by 40.7% (1 − e^−0.407^, *p* < 0.001), which means that de-skilling not only prolongs the working period but also reduces their workloads. This also means that de-skilling in the labor process not only prolongs working hours but also forces de-skilled workers to work overtime, and the regression results verify Hypothesis 1a. The reason for this phenomenon is that, firstly, de-skilling results in the loss of the employees’ technical advantages and is usually accompanied by a well-designed compensation system. The employees fall into a disadvantageous position with respect to employers, becoming “partial workers” who can be replaced at any time. Moreover, they have to compromise when employers force them to work overtime (Nie & Feng, 2020). Secondly, de-skilling is often accompanied by meticulously designed compensation systems, where flexible piece-rate mechanisms and fluctuating production quotas drive de-skilled workers, making employee overtime a routine component of corporate operations (Lv & Chen, 2014).

#### 4.2.2. The Impact of Labor “De-Skilling” on Employees’ Cognition of Overtime

Overtime cognition is related to both objective overtime work and the subjective recognition of overtime work by workers. Unconscious overtime work is defined as the workers’ inability to recognize that they are working overtime, while conscious overtime work is defined by the employees’ ability to recognize that they are indeed working overtime. The regression results are presented in Table 4.

From the regression results of the baseline model, it can be observed that, among personal characteristics, age, gender, education level, and income significantly affect employees’ overtime awareness. The probability of incurring unconscious overtime work increases by 1.6% (e^0.016^ − 1, *p* < 0.05) for every one-year increase in the age of the worker. Compared to male employees, female employees are more likely to work overtime unconsciously. The higher the education level, the more aware the individual is of their overtime behavior. Similarly to the overtime work variable, the probability of unconscious overtime work occurring for workers with a college education and above is 0.67 times lower than that of workers with education levels below college levels (1 − e^−0.394^, *p* < 0.001). The higher the employee’s income, the lower the probability of unconscious overtime occurring. The enterprise characteristic variables—labor union, labor contract, and the provision of food and accommodation variables—all have an impact on employees’ overtime awareness and are significant at the 0.1% level. Specifically, the probability of unconscious overtime work decreases by 29.8% (1 − e^−0.354^, *p* < 0.001) if the enterprise or unit has a labor union. Employees who have signed a labor contract are more likely to be aware of their overtime work. Employees are less likely to engage in unconscious overtime when employers provide on-site accommodation and meals.

The labor control model adds the labor de-skilling variable to the baseline model, and the regression results show that the significance of and coefficient changes in the control variables are low compared to the baseline model. Moreover, the labor de-skilling independent variable plays a significant role in the unconscious overtime of employees. The probability of unconscious overtime occurring is significantly higher for employees who are “de-skilled” at work, by 43.6% (e^0.362^ − 1, *p* < 0.001), compared to those who are not “de-skilled” at work. This also means that de-skilling in the labor process not only forces workers to extend working hours but also blurs the boundary between normal working hours and excess working hours, prompting low-skilled workers to work overtime unconsciously. This is the most important factor to be considered in the regression model, and the regression results verify Hypothesis 1b. De-skilling is often linked to the “piece-rate system”, in which variable output and fixed unit prices encourage employees to work longer hours to earn more; in addition, “de-skilled” subjects usually lack the corresponding awareness of rights and the concept of working hours, and overtime work is rationalized into normal working hours through the institutional design of the capital.

#### 4.2.3. The Impact of Labor “De-Skilling” on Employees’ Overtime Intensity

Focusing on the impact of the de-skilling of labor on the number of hours of overtime worked (intensity of overtime work), a multiple linear regression model with “objective hours of overtime worked” as a continuous variable was used, and the results of the regression are presented in Table 5.

From the regression results of the baseline model, it can be observed that, among the personal characteristics variables, only the role of income is not significant, and the rest of the variables play a significant effect on employees’ overtime work. Specifically, the older the employee, the shorter the overtime hours, and for every one-year increase in the age of the employee, the overtime hours are significantly reduced by 0.12 h (*p* < 0.001). Male individuals work longer overtime hours than female individuals by 1.69 h. The role of the domicile is also highlighted in the labor market, and the domicile segregation of the labor market is not only manifested in wages and income but also in working hours. Compared to employees with rural household registration, employees with urban household registration exhibit shorter overtime hours by 4.57 h (*p* < 0.001); employees with a high level of education have a lower probability of working overtime and shorter overtime hours. Moreover, a college degree or higher can help employees reduce their overtime hours by more than 5 h. The firm characteristic, unionization, labor contract, and provision of food and lodging variables all significantly affect the number of overtime hours worked by employees. Specifically, the signing of trade union and labor contracts is conducive to reducing the number of overtime hours worked by employees. Labor contracts protect the legal rights and interests of each employee at the legal level, including the working hours of employees. Employees whose workplaces provide accommodation and food experience a 3.5 h increase in overtime hours (*p* < 0.001).

From the regression results of the labor control model, it can be observed that the significance of the coefficient changes in the control variables is low compared to the baseline model, and the labor de-skilling independent variable has a significant effect on overtime work lengths. Employees who are “de-skilled” work significantly longer overtime hours by 1.34 h (*p* < 0.005)—that is, they experience more intensive overtime hours—and the regression results verify Hypothesis 1c.

### 4.3. The Mediating Role of Employees’ Job Autonomy

If job autonomy is influenced by labor de-skilling and, in turn, affects overtime behavior, this would demonstrate that job autonomy plays a mediating role. De-skilling not only directly increases working hours but also extends them indirectly by eroding job autonomy—forming a dual pathway where reduced control over work processes amplifies the overtime effect of de-skilling.

#### 4.3.1. The Role of Labor “De-Skilling” on Employees’ Job Autonomy

Table 6 shows the regression results with respect to employee job autonomy. The independent variable is labor de-skilling, and the dependent variable is employee job autonomy. The larger the value, the higher the employees’ job autonomy.

The core labor de-skilling independent variable exerts a significant negative effect on job autonomy; that is, employees experiencing de-skilling at their jobs have a significant reduction in job autonomy relative to 0.12 units (*p* < 0.05). The de-skilling of the labor process cuts employees’ work into fine-grained, simple, and repetitive tasks. In particular, the work autonomy of assembly line workers naturally decreases; however, independent developers, freelance designers, and other individuals who can autonomously complete work related to complex products tend to have a super-high degree of work autonomy. This shows that the de-skilling of the labor process has indeed reduced the power of employees at work and decreased their work autonomy.

#### 4.3.2. The Mediating Effect of Job Autonomy on Employees’ Willingness to Work Overtime

In order to test the possible indirect effect of reduced job autonomy induced by labor de-skilling on employees’ voluntary overtime, this study used a bootstrap self-help method to carry out sampling 500 in order to test this mediating effect. Table 7 demonstrates the direct and indirect effects of labor de-skilling, in which the direct effect is −0.083 and the indirect effect is −0.008; moreover, the confidence level of the results is 95%, and the mediating effect accounts for 8.5%. The results show that labor process de-skilling does not fully and directly affect employees’ voluntary overtime work; the reduction in employees’ work autonomy has a partial role to play in this phenomenon. Moreover, de-skilled employees have almost no free decision-making power regarding overtime work, which verifies Hypothesis 2a.

#### 4.3.3. The Mediating Effect of Job Autonomy on Employees’ Cognition of Overtime Work

In order to test the possible indirect effect of reduced job autonomy induced by labor de-skilling on employees’ perceptions of overtime, this study used the bootstrap self-help method, carrying out sampling 500 times to test this mediating effect. Table 8 demonstrates the direct and indirect effects of labor de-skilling, with a direct effect of 0.082 and an indirect effect of −0.001. However, this result is not significant, and Hypothesis 2b was not verified, which indicates that de-skilling in the labor process does not affect employees’ unconscious overtime behavior by reducing job autonomy and that job autonomy does not have a mediating effect on the relationship between labor de-skilling and employees’ unconscious overtime behavior. Instead, a mediating effect exists.

#### 4.3.4. The Mediating Effect of Job Autonomy on the Overtime Intensity of Employees

In order to test the possible indirect effect of reduced job autonomy caused by labor de-skilling on employees’ overtime intensity, this study used the bootstrap self-help method to carry out sampling 500 times in order to test this mediating effect. Table 9 demonstrates the direct and indirect effects of labor de-skilling, in which the direct effect is 1.267 and the indirect effect is 0.076. Moreover, the confidence level of the results is 95%, and the mediating effect is 5.7%. The results show that the de-skilling of the labor process does not entirely inflict a direct effect on the overtime work length of employees, and part of this effect is realized by reducing the autonomy of employees. The longer the overtime work, the greater the intensity of overtime work, which verifies Hypothesis 2c.

### 4.4. The Moderating Effect of Employees’ Occupational Value Orientation

While the “technological control” of the labor process only pushes simple workers into passive overtime, the overtime behavior of unskilled and complex workers may be moderated by the employees’ professional values.

#### 4.4.1. The Moderating Effect of Occupational Value Orientation on Employees’ Willingness to Work Overtime

Table 10 demonstrates the moderating effect of employees’ occupational value orientation on their voluntary overtime work, examining the survival and developmental orientations of the employees’ occupational value orientation. The first column of the regression results presents the moderating effect of the developmental orientation, and the second column of the regression results presents the moderating effect of the survival orientation; the variables in the table are taken as decentered values.

It can be observed that the development orientation factor is a moderating variable for voluntary overtime work, while the survival orientation factor does not exert a moderating effect. The development orientation of employees reinforces differences in the willingness to work overtime due to de-skilling. Employees who experience de-skilling on the job are generally forced to work overtime, while employees who are not de-skilled have a higher probability of carrying out voluntary overtime. If there is no technical control but only bureaucratic control in the workplace, employees with higher skill levels and a higher degree of self-determination in the workplace will be motivated to work voluntary overtime for self-fulfillment and self-development.

#### 4.4.2. The Moderating Effect of Occupational Value Orientation on Employees’ Cognition of Overtime Work

Table 11 demonstrates the moderating effect of occupational value orientation on employees’ perceptions of overtime. According to the regression results, the developmental orientation factor is a moderating variable for employees’ unconscious overtime work; in contrast, the survival orientation factor does not exhibit a moderating effect. The employee’s development orientation reinforces the difference in overtime cognition due to de-skilling. Employees with a tendency to “de-skill” their work are more likely to work overtime unconsciously; that is, they are more likely to under-recognize that they are carrying out overtime. If low-skilled employees also have strong developmental values, they are more likely to work overtime unconsciously, mirroring the results of employers’ strategies to extend workers’ working hours.

#### 4.4.3. The Regulating Effect of Occupational Value Orientation on the Overtime Intensity of Employees

Table 12 shows the moderating effect of employees’ professional value orientation on their overtime intensity. The development orientation factor is a moderating variable for employees’ overtime intensity (hours), while the survival orientation factor does not exhibit a moderating effect. The employee development orientation reinforces differences in overtime hours due to de-skilling, that is, if there are both technological and bureaucratic controls inflicted on the employees’ work—namely, if the production process is based on directed and measured labor and management establishes a set of rules and procedures and a culture that drives employees to pursue their personal interests and the interests of the firm—the overtime hours worked by employees will be significantly longer, and the intensity of overtime work will be elevated. Employers typically use these two parallel mechanisms to control workers’ working hours.

The comprehensive career value orientation regression results with respect to overtime willingness, overtime cognition, and overtime intensity, and the development orientation factor play a significant role; in contrast, the survival orientation factor results are not significant. Thus, Hypothesis 3a is verified, while Hypothesis 3b does not pass the significance test. The reason why survival orientation is not significant may lie in the fact that highly skilled employees at higher hierarchical and income levels no longer need to worry about basic survival; instead, the pursuit of high-level career development is their main behavioral motive.

## 5. Discussion

A review of classic theoretical frameworks on labor control reveals distinct historical trajectories: In Marx’s theory of surplus value, overtime is positioned as the most primitive mechanism of capital control, extracting additional surplus labor beyond necessary working hours. Braverman’s “de-skilling” and “technical control” theories highlight that as work processes are standardized and employee autonomy erodes, overtime becomes a “rational” managerial strategy to offset the upper limits of technical efficiency—turning extended hours into a compensatory measure for productivity plateaus. Edwards’ concept of “bureaucratic control” demonstrates how overtime is institutionalized through rules like attendance systems, transforming it from ostensibly “voluntary” to “covertly compulsory” by embedding work extension into organizational norms. Burawoy’s “hegemonic control” further shows that enterprises use “speed-up games” tied to performance metrics and promotion incentives, enabling employees to internalize the “legitimacy” of overtime as a pathway to individual advancement, thus reproducing control through consent rather than coercion.

Within empirical research, previous analyses of overtime phenomena have largely focused on macro-level perspectives, giving more attention to the impact of labor regulations (T. Wang, 2016), industry characteristics (Hou & He, 2020), and so on. Some studies have found that overtime work is prevalent in labor-intensive industries due to heavy production tasks (Nie & Feng, 2020), and some have observed that there are significant differences in overtime work with respect to labor markets inside and outside the system; moreover, overtime work is regarded as a “culture” (J. Wang & Fang, 2022). The existing analysis of overtime behavior provides an important foundation for this study.

While these theories and practices provide “rational justifications” for overtime, their essence remains capital’s control over labor time. Bureaucratic overtime regimes—embodied in attendance systems, performance metrics, and other institutional rules—present a veneer of fairness and efficiency, yet they obscure capital’s extraction of surplus value (Weber, 1922). Even when employees appear to “voluntarily” work overtime, this choice is often “structurally constrained voluntarism” (Bowles & Gintis, 1976), shaped by employment pressures and promotion competitions, such “voluntariness” masks the covert nature of labor control, where consent becomes a tool for reproducing exploitation.

Based on previous macro-analyses of the overtime phenomenon, this study examines the individual psychological and behavioral levels more in-depth. When overtime work becomes an objective reality, there is a difference in the workers’ perceptions of overtime work occurrences. Generally, by default, workers have a clear perception of overtime work; however, their perceptions may not be the same. Unconscious overtime is prevalent in the current labor market, which mostly occurs in low-skilled workers. Therefore, research on overtime work types should go beyond the existing classification. Moreover, whether workers are working overtime unconsciously should be determined based on their knowledge of overtime work and on the basis of whether they are actually working overtime or not.

Existing overtime research usually examines voluntary or involuntary overtime work and overtime compensation, and it is believed that voluntary overtime work can be exchanged for promotion opportunities and financial compensation (Lv & Chen, 2014; Cai & Shi, 2016). However, this study on unconscious overtime work expands the analysis of overtime behavior beyond this extent. In particular, this study places special attention on the influence of individual employees’ work values, career development expectations, and other factors with respect to overtime behavior. This is different from previous explanations of overtime behavior, which proceed solely from the perspective of the external environment. Overtime behavior is not only affected by external pressures, but is also closely related to employees’ own intrinsic motivations.

Contemporary labor studies must urgently address emerging labor control phenomena and mechanisms including digital surveillance systems that leverage time-tracking software, log overtime hours, and enforce compliance with technical efficiency benchmarks, effectively obligating employees to work extended hours to meet algorithmically defined productivity standards. Algorithmic control in gig economies, as exemplified by food delivery platforms using algorithms to shrink delivery timeframes, compels riders into overtime to maintain income—a form of work intensification that masks exploitative surplus extraction. Technological domination, manifested in the spatial-temporal penetration of instant communication tools, enables enterprises to blur work–life boundaries through these technologies, generating “covert overtime”—unofficial extended work hours imposed by digital connectivity. Institutional incentive structures, embedded in “project bonus” schemes and tacitly coercive compensation models, induce voluntary overtime by framing extra work as a prerequisite for career progression or performance-based rewards.

Cultural internalization of work norms, as conceptualized by Kunda (1992), involves corporate discourses that construct overtime as a pathway to “self-actualization”, leading employees to perceive extended hours as a personal responsibility rather than a systemic demand. Institutional isomorphism (DiMaggio & Powell, 1983) normalizes overtime through industry-wide narratives of “competitive necessity” or “project urgency”, embedding excessive work hours into organizational legitimacy frameworks. Confronting these multifaceted overtime practices requires collective bargaining and union engagement: by negotiating labor quotas through collective contracts, stakeholders can establish structural safeguards against disguised overtime perpetuated by technological imperatives or cultural normativization.

## 6. Conclusions

Based on data from the China Labor Force Dynamics Survey, this study discusses employee overtime behaviors from the perspective of labor process theory; namely, assessing the effects of labor de-skilling on overtime willingness, overtime cognition, and overtime intensity. The specific findings are as follows: Technical control in the labor process has a direct effect on employees’ working hours, and de-skilling significantly prolongs employees’ overtime hours, resulting in the provision of overtime consent and the phenomenon of unconscious overtime. De-skilling in the labor control process also reduces employees’ decision-making autonomy, resulting in voluntary overtime and prolonged working hours. In addition, capital has a significant impact on employees’ willingness to work overtime and overtime intensity. In addition, capital’s control of labor is not only achieved through de-skilling but also through bureaucratic control, shaping career development values in order to both increase overtime intensities by extending working hours and reduce costs by lowering the level of resistance.

The analysis showed that the de-skilling of the labor process is distinguished between de-skilled and complex workers; moreover, depending on capital, different control methods for different types of workers are developed. De-skilled workers are mostly employed in traditional manufacturing industries and low-end service industries, in which scientific management methods are adopted to reduce the wages of workers and extend their working hours through the streamlining and standardization of the production process and the package accommodation system. As for highly skilled workers (managers and professionals), capital adopts human capital theories based on self-realization, self-investment, and self-development to promote the self-management of complex workers. These methods result in the perception that opportunities for learning and improvement are provided—rather than the presence of exploitation—which is a kind of “honor” (Hewlett & Carolyn, 2007).

Due to the limitations of the research data, this study only focused on the impact of de-skilling (or not) on the working hours of employees; moreover, it failed to pay attention to the differences in the factors affecting overtime work between different occupations and different industries. In addition, this study used a quantitative analysis approach to explore overtime issues under labor control, inevitably falling into the trap of absolutism by measuring the intensity of overtime work in terms of working hours, thus ignoring special and common overtime phenomena such as “fishing” and “inward roll” occurrences. Overtime work may be the result of labor control by employers, but may also be employed as a means of resistance by workers: in non-piece-rate forms of labor, workers are “grinding” to cope with their work. It is always important to examine labor–management conflicts in a reasonable manner and to try to explore effective methods for harmonizing labor–management relations.

We hope that future research will further expand on the relationship between overtime work and health, the effects of overtime behaviors in work–family relationships, and gender differences in overtime behaviors. Specifically, the effects of overtime work on physical health (e.g., fatigue, cardiovascular disease, and other physical symptoms) should be focused on, and mental health consequences (e.g., anxiety, depression, burnout, and other psychological symptoms) should also be examined. Moreover, the following should also be investigated in order to further enrich the body of research focused on overtime: the manner in which overtime work affects the fulfillment of employees’ roles in the family and how overtime work affects the quality of family relationships, as well as the differences in overtime work in terms of time management skills, role adjustments, and psychological adjustment between different genders.

## Figures and Tables

**Table 1:** Definitions of variables and descriptive statistics.

Variable Name	Variable Type	Mean/Percent	Standard Deviation	Indicates	Sample Size
Overtime hours	Continuous variable	8.20	12.19	Weekly overtime (h) Value range: [0, 120]	5263
Objective overtime	Binary categorical variable	No = 47.7%; Yes = 52.3%	0.500	0 = no; 1 = yes	5263
Subjective overtime	Binary categorical variable	No = 67.4%; Yes = 32.6%	0.469	0 = no; 1 = yes	5263
Voluntary overtime	Binary categorical variable	No = 35.7%; Yes = 64.3%	0.479	0 = no; 1 = yes	1719
Unconscious overtime	Binary categorical variable	No = 37.9%; Yes = 62.1%	0.485	0 = no; 1 = yes	2750
Age	Continuous variable	40.25	10.938	Value range: [18, 64]	5263
Sex	Binary categorical variable	Female = 44.6%; Male = 55.4%	0.497	0 = female; 1 = male	5263
Educational level	Binary categorical variable	College degree or below = 68.8%; College and above = 31.2%	0.464	0 = junior college or below; 1 = college degree or above	5263
Household registration	Binary categorical variable	Rural = 58.9%; Town = 47.1%	0.499	0 = rural; 1 = town	5263
Trade unionist	Binary categorical variable	No = 66.9%; Yes = 33.1%	0.470	0 = no; 1 = yes	5263
Labor contract	Binary categorical variable	No = 46.8%; Yes = 53.2%	0.499	0 = no; 1 = yes	5263
Provide accommodation	Binary categorical variable	No = 83.3%; Yes = 16.7%	0.373	0 = no; 1 = yes	5263
Job autonomy	Continuous variable	5.15	1.988	Value range: [3, 9]	5263
Annual income (logarithm)	Continuous variable	9.59	2.781	Value range: [0, 15]	5263
Development orientation	Continuous variable	61.24	17.029	Value range: [0, 100]	5263
Survival orientation	Continuous variable	72.97	14.253	Value range: [0, 100]	5263

**Table 2:** Types of overtime work carried out by employees (unit: %).

	Objective Overtime	Subjective Overtime	Voluntary Overtime	Unconscious Overtime
Yes	52.25	32.64	64.34	62.15
No	47.75	67.36	35.66	37.85
Total	100 (5263)	100 (5263)	100 (1719)	100 (2750)

**Table 3:** Regression results of labor de-skilling affecting employees’ willingness to work overtime.

Variable		Reference Model	Labor Control Model
De-skilling of labor			−0.407 ***
			(0.114)
Personal characteristics	Age	−0.006	−0.005
		(0.005)	(0.005)
	Gender (male = 1)	−0.037	−0.075
		(0.105)	(0.106)
	Household registration (town = 1)	−0.275 *	−0.303 *
		(0.123)	(0.125)
	Education level (college or above = 1)	0.147	0.094
		(0.131)	(0.133)
	Income	0.061 **	0.059 **
		(0.019)	(0.020)
Enterprise characteristics	Union (have = 1)	−0.245 *	−0.298 *
		(0.117)	(0.118)
	Labor contract (have = 1)	0.0314	−0.0412
		(0.114)	(0.116)
	Accommodation available (Yes = 1)	0.419 **	0.432 **
		(0.136)	(0.136)
Constant		0.323	0.562 +
		(0.288)	(0.301)
Pseudo R^2^		0.0230	0.0174
Observations		1719	1719

Note: Standard error in parentheses. *** *p* < 0.001; ** *p* < 0.01; * *p* < 0.05; + *p* < 0.1.

**Table 4:** Regression results of labor de-skilling affecting employees’ cognition of overtime work.

Variable		Reference Model	Labor Control Model
De-skilling of labor			0.362 ***
			(0.087)
Personal characteristics	Age	0.0160 ***	0.0142 ***
		(0.004)	(0.004)
	Gender (male = 1)	−0.138 +	−0.106
		(0.084)	(0.084)
	Household registration (town = 1)	−0.0356	−0.0193
		(0.097)	(0.097)
	Education level (college or above = 1)	−0.394 ***	−0.345 **
		(0.119)	(0.119)
	Income	−0.0256 +	−0.0222
		(0.015)	(0.015)
Enterprise characteristics	Union (have = 1)	−0.354 ***	−0.297 **
		(0.102)	(0.103)
	Labor contract (have = 1)	−0.494 ***	−0.430 ***
		(0.088)	(0.090)
	Accommodation available (yes = 1)	−0.347 ***	−0.350 ***
		(0.099)	(0.100)
Constant		0.680 **	0.463 *
		(0.217)	(0.223)
Pseudo R^2^		0.046	0.051
Observations		2750	2750

Note: Standard error in parentheses. *** *p* < 0.001; ** *p* < 0.01; * *p* < 0.05; + *p* < 0.1.

**Table 5:** Regression results of labor de-skilling affecting employees’ overtime intensity.

Variable		Reference Model	Labor Control Model
De-skilling of labor			1.343 **
			(0.492)
Personal characteristics	Age	−0.120 ***	−0.127 ***
		(0.025)	(0.025)
	Gender (male = 1)	1.690 ***	1.803 ***
		(0.469)	(0.469)
	Household registration (town = 1)	−4.749 ***	−4.68 ***
		(0.553)	(0.554)
	Education level (college or above = 1)	−5.308 ***	−5.129 ***
		(0.542)	(0.547)
	Income	0.052	0.064
		(0.102)	(0.102)
Enterprise characteristics	Union (have = 1)	−1.546 **	−1.340 **
		(0.503)	(0.509)
	Labor contract (have = 1)	−1.060 *	−0.840 +
		(0.492)	(0.493)
	Accommodation available (yes = 1)	3.495 ***	3.503 ***
		(0.717)	(0.716)
Constant		11.45 ***	10.64 ***
		(1.392)	(1.429)
Pseudo R^2^		0.086	0.087
Observations		5263	5263

Note: Standard error in parentheses. *** *p* < 0.001; ** *p* < 0.01; * *p* < 0.05; + *p* < 0.1.

**Table 6:** Regression results of labor de-skilling affecting employees’ job autonomy.

Variable	Job Autonomy Model
De-skilling of labor	0.120 *
	(0.059)
Individual and firm characteristic variables	Controlled
Constant	5.525 ***
	(0.157)
Observations	5263
R-squared	0.022

Note: Standard error in parentheses. *** *p* < 0.001; * *p* < 0.05.

**Table 7:** Mediating effect test of the influence of job autonomy on employees’ willingness to work overtime.

Type	Coefficient	Self-Service Standard Error	Z-Value	*p*-Value	95% Confidence Interval	
Indirect effect	−0.008	0.004	−1.96	0.050	−0.016	−0.001
Direct effect	−0.083	0.025	−3.19	0.001	−0.137	−0.034
Total effect	−0.091	0.026	−3.55	0.000	−0.141	−0.041
Proportion of mediating effect	8.5%					

**Table 8:** Mediating effect test of job autonomy on employees’ cognition of overtime work.

Type	Coefficient	Self-Service Standard Error	Z-Value	*p*-Value	95% Confidence Interval	
Indirect effect	−0.001	0.001	−1.33	0.183	−0.004	0.000
Direct effect	0.082	0.199	4.23	0.000	0.045	0.125
Total effect	0.081	0.195	4.16	0.000	0.044	0.120
Proportion of mediating effect	−1.7%					

**Table 9:** Mediating effect tests of the influence of job autonomy on the overtime intensity of employees.

Type	Coefficient	Self-Service Standard Error	Z-Value	*p*-Value	95% Confidence Interval	
Indirect effect	0.076	0.042	1.80	0.048	0.007	0.159
Direct effect	1.267	0.498	2.54	0.011	0.287	2.245
Total effect	1.343	0.499	2.69	0.007	0.364	2.321
Proportion of mediating effect	5.7%					

**Table 10:** The moderating effect of occupational value orientation on employees’ willingness to work overtime.

Variable		Voluntary Overtime	
Control Variable		Controlled	
Independent variable	De-skilling of labor	−0.365 **	−0.346 **
		(0.115)	(0.116)
Occupational value orientation	Development orientation	0.016 ***	0.012 ***
		(0.004)	(0.003)
	Survival orientation	−0.0001	−0.004
		(0.004)	(0.005)
Interaction term	Labor de-skilling × development orientation factor	−0.010 +	
		(0.006)	
	Labor de-skilling × survival-oriented factor		0.009
			(0.007)
Constant		−0.418	0.098
		(0.465)	(0.477)
Observations		1719	1719
Pseudo R^2^		0.0312	0.0317

Note: Standard error in parentheses. *** *p* < 0.001; ** *p* < 0.01; + *p* < 0.1.

**Table 11:** The moderating effect of occupational value orientation on employees’ cognition of overtime work.

Variable		Voluntary Overtime	
Control Variable		Controlled	
Independent variable	De-skilling of labor	0.397 ***	0.381 ***
		(0.088)	(0.088)
Occupational value orientation	Development orientation	−0.001	0.004
		(0.003)	(0.002)
	Survival orientation	−0.006 *	−0.008 +
		(0.003)	(0.004)
Interaction term	Labor de-skilling × development orientation factor	0.010 *	
		(0.005)	
	Labor de-skilling × survival-oriented factor		0.004
			(0.006)
Constant		0.975 **	0.805 *
		(0.367)	(0.391)
Observations		2750	2750
Pseudo R^2^		0.054	0.053

Note: Standard error in parentheses. *** *p* < 0.001; ** *p* < 0.01; * *p* < 0.05; + *p* < 0.1.

**Table 12:** Test of the moderating effect of occupational value orientation on the overtime intensity of employees.

Variable		Voluntary Overtime	
Control Variable		Controlled	
Independent variable	De-skilling of labor	1.123 *	1.130 *
		(0.496)	(0.497)
Occupational value orientation	Development orientation	−0.068 **	−0.034 *
		(0.021)	(0.015)
	Survival orientation	0.087 ***	0.073 ***
		(0.016)	(0.020)
Interaction term	Labor de-skilling × development orientation factor	0.067 *	
		(0.031)	
	Labor de-skilling × survival-oriented factor		0.027
			(0.033)
Constant		8.953 ***	7.700 ***
		(2.130)	(2.078)
Observations		5263	5263
Pseudo R^2^		0.094	0.093

Note: Standard error in parentheses. *** *p* < 0.001; ** *p* < 0.01; * *p* < 0.05.

## Data Availability

The data that support the findings of this study can be applied via the official e-mail address of the China Labor-force Dynamics Survey (cssdata@mail.sysu.edu.cn).
